# MiR‐17‐3p inhibits osteoblast differentiation by downregulating *Sox6* expression

**DOI:** 10.1002/2211-5463.12979

**Published:** 2020-10-25

**Authors:** Nan Chen, Di Wu, Hua Li, Yi Liu, Hao Yang

**Affiliations:** ^1^ Department of Orthopedics the First Affiliated Hospital of Kunming Medical University Kunming China

**Keywords:** MC3T3‐E1, miR‐17‐3p, osteoblast, *Sox6*

## Abstract

Osteoporosis and osteoarthritis are orthopedic disorders that affect millions of elderly people worldwide; stimulation of bone formation is a potential therapeutic strategy for the treatment of these conditions. As the only bone‐forming cells, osteoblasts play a key role in bone reconstruction. The microRNA miR‐17‐3p is downregulated during osteogenic differentiation of human bone marrow mesenchymal stem cells, but its precise role in this process is unknown. Here, we investigated the role of miR‐17‐3p in osteoblast differentiation. An *in vitro* model of osteogenesis was established by treating MC3T3‐E1 murine preosteoblast cells with bone morphogenetic protein 2 (BMP2). The expression of miR‐17‐3p in BMP2‐induced MC3T3‐E1 cells was detected by reverse transcription‐quantitative PCR, and its effects on cells transfected with miR‐17‐3p mimic or inhibitor were evaluated by Alizarin Red staining, alkaline phosphatase (ALP) activity assay, and by detection of osteoblast markers including the ALP, collagen type I α1 chain, and osteopontin genes. Bioinformatics analysis was carried out to identify putative target genes of miR‐17‐3p, and the luciferase reporter assay was used for functional validation. Rescue experiments were performed to determine whether SRY‐box transcription factor 6 (*Sox6*) plays a role in the regulation of osteoblast differentiation by miR‐17‐3p. We report that miR‐17‐3p was downregulated upon BMP2‐induced osteoblast differentiation in MC3T3‐E1 cells, and this was accompanied by decreased differentiation and mineralization, ALP activity, and expression of osteogenesis‐related genes. *Sox6* was confirmed to be a target gene of miR‐17‐3p in osteoblasts, and the inhibitory effect of miR‐17‐3p on osteoblast differentiation was observed to occur via *Sox6*. These results suggest the existence of a novel mechanism underlying miRNA‐mediated regulation of osteogenesis, which has potential implications for the treatment of orthopedic disorders.

## Graphical abstract

An *in vitro* model of osteogenesis was established by treating MC3T3‐E1 murine preosteoblast cells with BMP2. MiR‐17‐3p inhibited the differentiation of MC3T3‐E1 cells. *Sox6* was confirmed to be a target gene of miR‐17‐3p, and the inhibitory effect of miR‐17‐3p on osteoblast differentiation was reversed by *Sox6*. These results suggest potential implications for the treatment of orthopedic disorders.

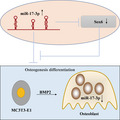

**Abbreviations**
ALP: alkaline phosphatase
BMP2: bone morphogenetic protein 2
COLIA1: collagen type I α1 chain
GAPDH: glyceraldehyde‐3‐phosphate dehydrogenase
miR‐17‐3p: microRNA‐17‐3p
miRNA: microRNA
MUT: mutation‐type
NC: negative control
OPN: osteopontin
RT‐qPCR: reverse transcription‐quantitative PCR
SD: standard deviation
SiRNA: Small interfering RNA
Sox6: SRY‐box transcription factor 6
UTR: untranslated region
WT: wild‐type

Osteoporosis and osteoarthritis are orthopedic disorders that affect millions of elderly people worldwide and are characterized by bone nonunion, loss, and defects that lead to impaired bone formation and bone deterioration [1]. Stimulating bone formation is a potential therapeutic strategy for the treatment of these conditions. As the only bone‐forming cells, osteoblasts play a key role in bone reconstruction [2]. Bone formation involves the differentiation of progenitor cells into osteoblasts; inhibiting this process can have pathologic consequences [3]. Osteogenic induction increases the expression of osteogenesis‐related genes including osteopontin (OPN), collagen type I α1 chain (COLIA1), and alkaline phosphatase (ALP) [4], which is a key event in osteoblast differentiation.

MicroRNAs (miRNAs) regulate a variety of cellular processes, and their dysregulation has been implicated in several diseases. Their activity mainly involves binding to the 3′ untranslated region (UTR) of target transcripts to alter gene expression [5, 6]. The role of miRNAs in osteogenesis and bone development has been widely investigated. Multiple miRNAs including miR‐138, miR‐2861, and miR‐148b have been shown to modulate the development of bone precursor cells [7, 8, 9]. MiR‐17‐3p is downregulated during osteogenic differentiation of human bone marrow mesenchymal stem cells [10], but its precise role in this process is unknown.

Transcription factors such as Runt‐related transcription factor 2 (Runx2), Osterix, mothers against DPP homolog 1 (SMAD), T‐cell factor (TCF)/Lymphoid enhancer‐binding factor (LEF), nuclear factor of activated T‐cell cytoplasmic 1 (NFATc1), Twist, activator protein 1 (AP‐1), and activating transcription factor 4 (ATF4) are known to play an important role in osteogenic differentiation [11, 12]. The transcription factor SRY‐box transcription factor 6 (Sox6) is involved in the differentiation of various tissues [13]. It was previously reported that *Sox6* is a tumor suppressor gene that is downregulated in osteosarcoma (OS) tissues and cell lines. Osteogenic differentiation defects promote the development of OS; therefore, stimulating this process is a potential treatment strategy [14].

Based on the above findings, the present study investigated the roles of miR‐17‐3p and *Sox6* in osteoblast differentiation in order to assess their potentiality as therapeutic targets in the treatment of bone disorders.

## Materials and methods

### Cell culture

MC3T3‐E1 murine preosteoblast cells were obtained from ScienCell (Carlsbad, CA, USA) and cultured in α‐minimal essential medium supplemented with 10% fetal bovine serum, penicillin (100 U·mL^−1^), and streptomycin (100 Ig·mL^−1^) at 37 °C and 5% CO_2_. Cells in passages 2 and 3 were used for experiments. To induce osteoblast differentiation, the cells were cultured in medium containing 200 ng·mL^−1^ BMP2 for 21 days.

### Transfection of MC3T3‐E1 cells

MiR‐17‐3p mimic and miR‐17‐3p inhibitor, along with small interfering RNA (siRNA) targeting *Sox6* or a scrambled control siRNA, were synthesized by GenePharma (Shanghai, China). The plasmids were transfected into MC3T3‐E1 cells at a concentration of 10 nm using Lipofectamine RNAiMAX transfection reagent (cat. no. 13‐778‐075; Invitrogen, Carlsbad, CA, USA) according to the manufacturer’s protocol.

### Real‐time quantitative PCR

After osteoinduction of MC3T3‐E1 cells for 0, 1, 3, 7, 14, and 21 days, total RNA was extracted from cells using TRIzol reagent (GenePharma). cDNA was synthesized from 500 ng total RNA using the Bestar qPCR RT kit (DBI Bioscience, Shanghai, China), and 20–100 ng of cDNA served as the template for RT‐qPCR using Bestar SybrGreen qPCR Master Mix (DBI Bioscience) according to the manufacturer’s protocol. MiR‐17‐3p and *Sox6* levels and the transfection efficiency of miR‐17‐3p mimic or inhibitor in MC3T3‐E1 cells were determined by RT‐qPCR. The expression of osteogenic differentiation‐related genes was also detected by RT‐qPCR in 3 independent experiments. Primers used for RT‐qPCR are shown in Table 1. U6 and glyceraldehyde 3‐phosphate dehydrogenase (GAPDH) served as internal controls for miRNA and mRNA, respectively. Relative expression levels of target genes were calculated with the 2^−ΔΔCt^ method, with *GAPDH* level used for normalization.

**Table 1:** All primers used in qPCR.

Gene	Gene ID	Transcript ID	Forward primer (5'‐3')	Reverse primer (5'‐3')	Loci	Transcript length	Amplification length	Annealing temperature
Sox6	20679	NM_011445.4	GAGCCCAGGTTTGTCTCCATC	CCAGCGAGGAAGAGAAATTGC	6122–6334	8715	213	54.9, 55.5
ALP	11647	NM_007431.3	CTACGCACCCTGTTCTGAGG	GGCCAAAGGGCAATAACTAG	1801–2020	2524	220	52.2, 50.9
COLIA1	12842	NM_007742.4	GAAGCTTGGTCCTCTTGCTTG	CATTGCCTTTGTTTGCTGGG	4783–4980	5946	198	53.3, 55.5
OPN	20750	NM_001204201.1	GGACTGAGGTCAAAGTCTAGGAG	GGAATGCTCAAGTCTGTGTG	606–839	1475	234	50.7, 47.6
GAPDH	14433	NM_001289726.1	CATCATCCCTGCATCCACTG	CAACCTGGTCCTCAGTGTAG	701–930	1296	230	53.4, 46.7
miR‐17‐3P	723905	MI0000687	GCTCTGAUGUUCACGGAAGUG	GTGCAGGGTCCGAGGT	51–72	84	70	52.2, 47.7
U6	19862	NR_003027.2	CTCGCTTCGGCAGCACATATAC	GGAACGCTTCACGAATTTGC	4–	107	96	56.4, 54.7

### Western blot analysis

Total protein was extracted using radioimmunoprecipitation assay buffer (pH 7.4) and separated by sodium dodecyl sulfate–polyacrylamide gel electrophoresis on a 10% gel. The proteins were transferred over 2 h to a nitrocellulose membrane (Millipore, Billerica, MA, USA) that was then blocked with 5% low‐fat milk at room temperature for 2 h and incubated overnight with a primary antibody against Sox6 (1:1000, cat. no. ab66316; Abcam, Cambridge, MA, USA), followed by horseradish peroxidase‐conjugated secondary antibody (1 : 5000, goat anti‐rabbit IgG H&L, cat. no. ab205718; Abcam) for 2 h at room temperature. Protein bands were detected with enhanced chemiluminescent reagent (Amersham Biosciences, Piscataway, NJ, USA). GAPDH was used as the loading control.

### ALP activity and Alizarin Red staining

ALP activity was detected to assess the degree of differentiation of MC3T3‐E1 cells. After transfecting BMP2‐treated MC3T3‐E1 cells with miR‐17‐3p mimic or inhibitor or siRNA, ALP activity was determined using a commercial assay kit (Jiancheng Biotech, Nanjing, China), with absorbance measured at 405 nm. Calcification in MC3T3‐E1 cells was also detected by staining with Alizarin Red for 30 min at room temperature, and the absorbance was measured at 540 nm using a microplate reader. Images of stained cells were acquired on a light microscope.

### Plasmid construction and dual‐luciferase activity assay

*Sox6* was predicted as a target gene of miR‐17‐3p using TargetScan (http://www.targetscan.org/vert_72/). The fragment of *Sox6* containing miR‐17‐3p binding sites was amplified by PCR with specific primers and cloned into the psi‐CHEK2 vector (Promega, Madison, WI, USA) to obtain wild‐type (WT) *Sox6* plasmid. A fragment containing mutated miR‐17‐3p‐binding sites was also amplified and inserted into psi‐CHEK2 to generate the mutant (MUT) *Sox6* plasmid. WT and MUT *Sox6* 3′ UTR DNA sequences were synthesized by GenePharma. MC3T3‐E1 cells incubated overnight in 24‐well plates were cotransfected with WT or MUT *Sox6* plasmid and miR‐17‐3p mimic or negative control (NC) using Lipofectamine 2000 (Invitrogen). Luciferase activity was determined with the Dual‐Luciferase Reporter Assay System (Promega) according to the manufacturer’s instructions, with absorbance measured at 560 nm.

### Statistical analysis

Statistical analysis was performed using prism v5.0 software (GraphPad, La Jolla, CA, USA). All data are presented as mean ± standard deviation (SD). Differences between groups were evaluated with the Wilcoxon test. The experiments were carried out independently three times. *P* < 0.05 was considered statistically significant.

## Results

### MiR‐17‐3p is downregulated during BMP2‐induced osteoblast differentiation

**Fig. 1:**
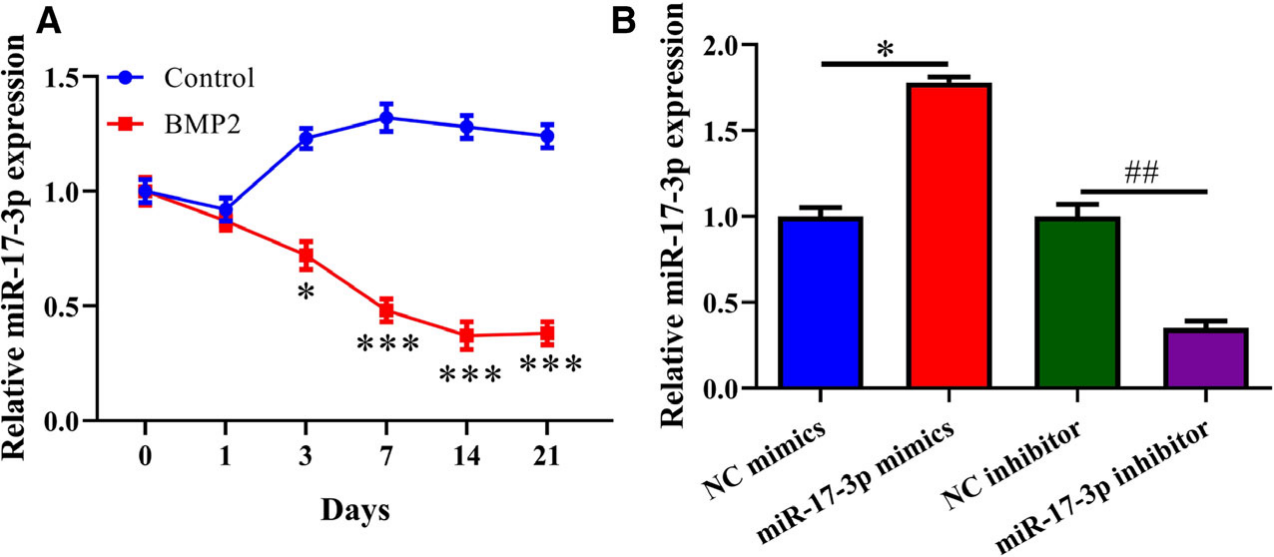
MiR‐17‐3p is downregulated during osteoblast differentiation. (A, B) miR‐17‐3p level in BMP2‐treated MC3T3‐E1 cells (A) and cells transfected with miR‐17‐3p mimic or inhibitor for 24 h (B), as determined by RT‐qPCR. The error bars indicate SD. Wilcoxon test was used to evaluate the differences between groups. *n* = 3, *P < 0.05, **P < 0.0, ^##^ *P* < 0.01.

MiR‐17‐3p expression levels in MC3T3‐E1 cells on days 0, 1, 3, 7, 14, and 21 of osteoblast differentiation were determined by RT‐qPCR. MiR‐17‐3p expression decreased over time in BMP2‐treated cells compared to the untreated control group (Fig. 1A). In MC3T3‐E1 cells transfected with miR‐17‐3p mimic or inhibitor, miR‐17‐3p level was higher and lower, respectively, than in cells transfected with NC, which confirmed the efficiency of transfection (Fig. 1B).

### MiR‐17‐3p attenuates osteoblast differentiation

**Fig. 2:**
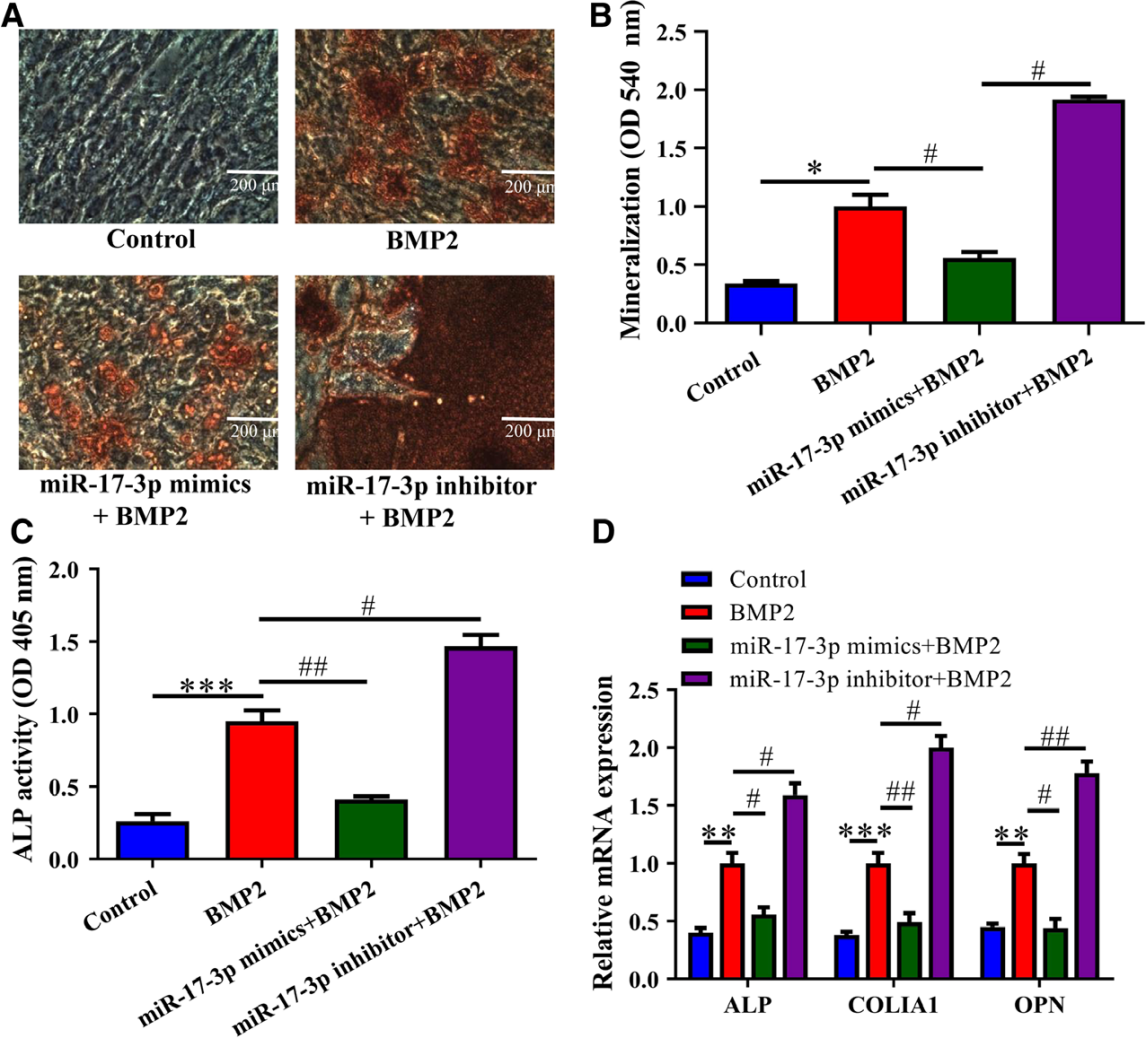
MiR‐17‐3p suppresses osteoblast differentiation in MC3T3‐E1 cells. Cells were transfected with miR‐17‐3p mimic or inhibitor and treated with 200 ng·mL^−1^ BMP2 for 14 days. Transfected cells without BMP treatment or without transfection but treated with BMP2 served as negative controls. (A) Calcification in cells detected by Alizarin Red staining, the length of the scale bars is 200 μm. (B) Matrix mineralization was evaluated by Alizarin Red staining. (C) ALP activity detected with a commercial assay kit. (D) mRNA levels of osteoblast marker genes (*ALP*, *COLIA1*, and *OPN*) determined by RT‐qPCR. The error bars indicate SD. Wilcoxon test was used to evaluate the differences between groups. *n* = 3, **P < 0.01, ***P < 0.001; ^#^ *P* < 0.05, ^##^ *P* < 0.01.

To examine the role of miR‐17‐3p in osteoblast differentiation, miR‐17‐3p mimic or inhibitor was transfected into MC3T3‐E1 cells following BMP2 induction. Overexpression of miR‐17‐3p significantly reduced calcification compared to BMP2‐treated NC‐transfected control cells, whereas inhibition of miR‐17‐3p had the opposite effect. Similar trends were observed for mineralization (Fig. 2A,B). Furthermore, compared to cells with BMP2 treatment, ALP activity was decreased in BMP2‐induced cells transfected with miR‐17‐3p mimic and increased in those transfected with miR‐409‐3p inhibitor (Fig. 2C), with corresponding decreases or increases in the expression levels of the osteoblast marker genes *ALP*, *COLIA1*, and *OPN* (Fig. 2D). These results confirm that miR‐17‐3p inhibits BMP2‐induced osteoblast differentiation in MC3T3‐E1 cells.

### *Sox6* is a target gene of miR‐17‐3p

**Fig. 3:**
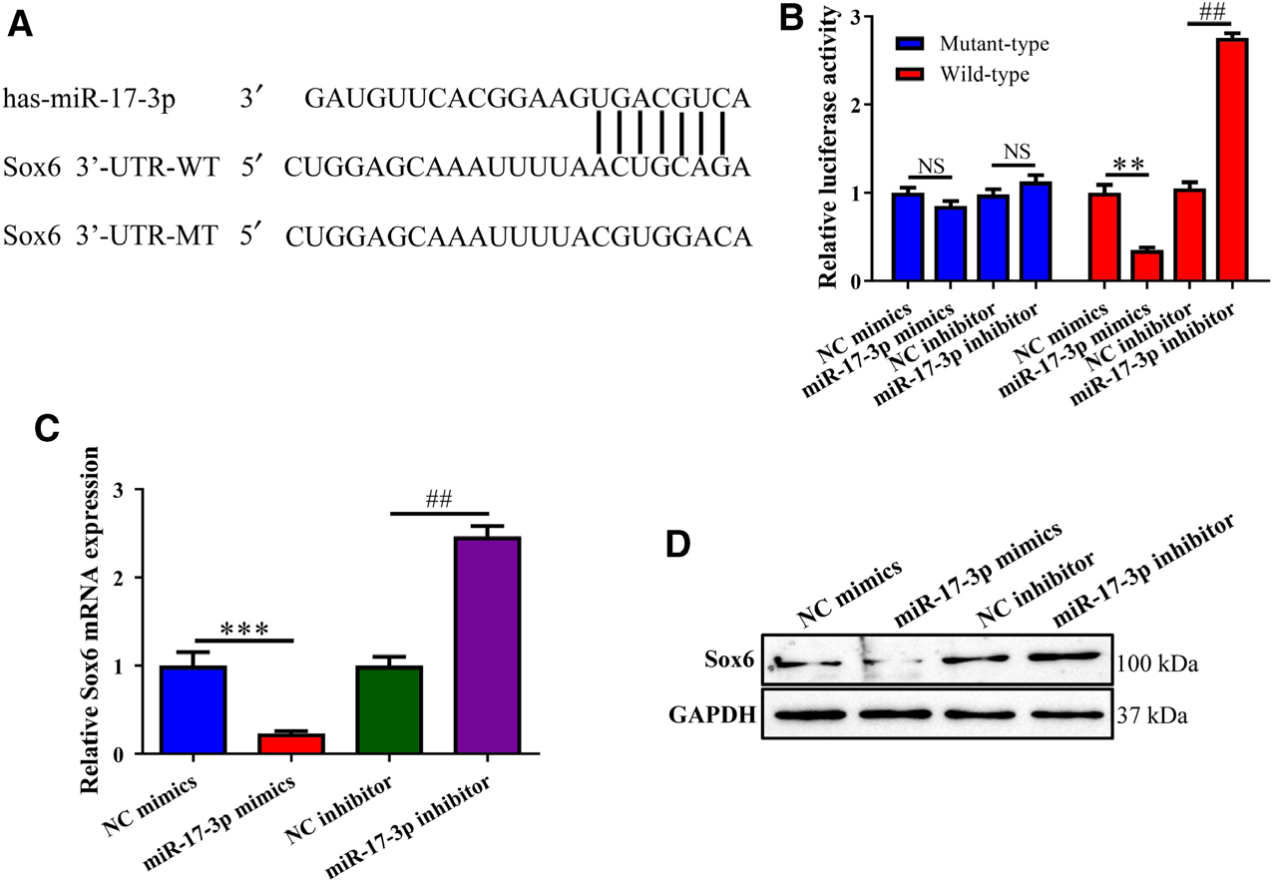
*Sox6* is a target gene of miR‐17‐3p in osteoblasts. (A) Sequence alignment of seed‐matched binding sites for miR‐17‐3p in the *Sox6* 3' UTR. (B) Effect of miR‐17‐3p on luciferase activity in MC3T3‐E1 cells transfected with the *Sox6* 3' UTR luciferase reporter detected 48‐h transfection. (C, D) *Sox6* mRNA (C) and protein (D) levels determined by RT‐qPCR and western blotting, respectively, following transfection with miR‐17‐3p mimic or miR‐17‐3p inhibitor for 24 h. The error bars indicate SD. Wilcoxon test was used to evaluate the differences between groups. *n* = 3, **P < 0.01, ***P < 0.001; ^##^ *P* < 0.01.

The TargetScan prediction algorithm identified putative miR‐17‐3p‐binding sites in the *Sox6* 3' UTR (Fig. 3A). We found that luciferase activity was reduced in MC3T3‐E1 cells cotransfected with miR‐17‐3p mimic and *Sox6* WT 3' UTR compared to those that were cotransfected with NC mimic + *Sox6* WT 3' UTR, whereas an increase in activity was observed in cells cotransfected with miR‐17‐3p inhibitor + *Sox6* MUT 3' UTR. On the other hand, there was no change in luciferase activity in cells cotransfected with miR‐17‐3p mimic or inhibitor and *Sox6* MUT 3' UTR (Fig. 3B). Furthermore, in cells transfected with miR‐17‐3p mimic, *Sox6* mRNA and protein levels were markedly reduced compared to cells transfected with NC mimic, as determined by RT‐qPCR and western blotting, respectively (Fig. 3C,D). These results demonstrate that *Sox6* expression in osteoblast differentiation is regulated by miR‐17‐3p.

### *Sox6* knockdown reverses the inhibition of osteoblast differentiation by miR‐17‐3p

**Fig. 4:**
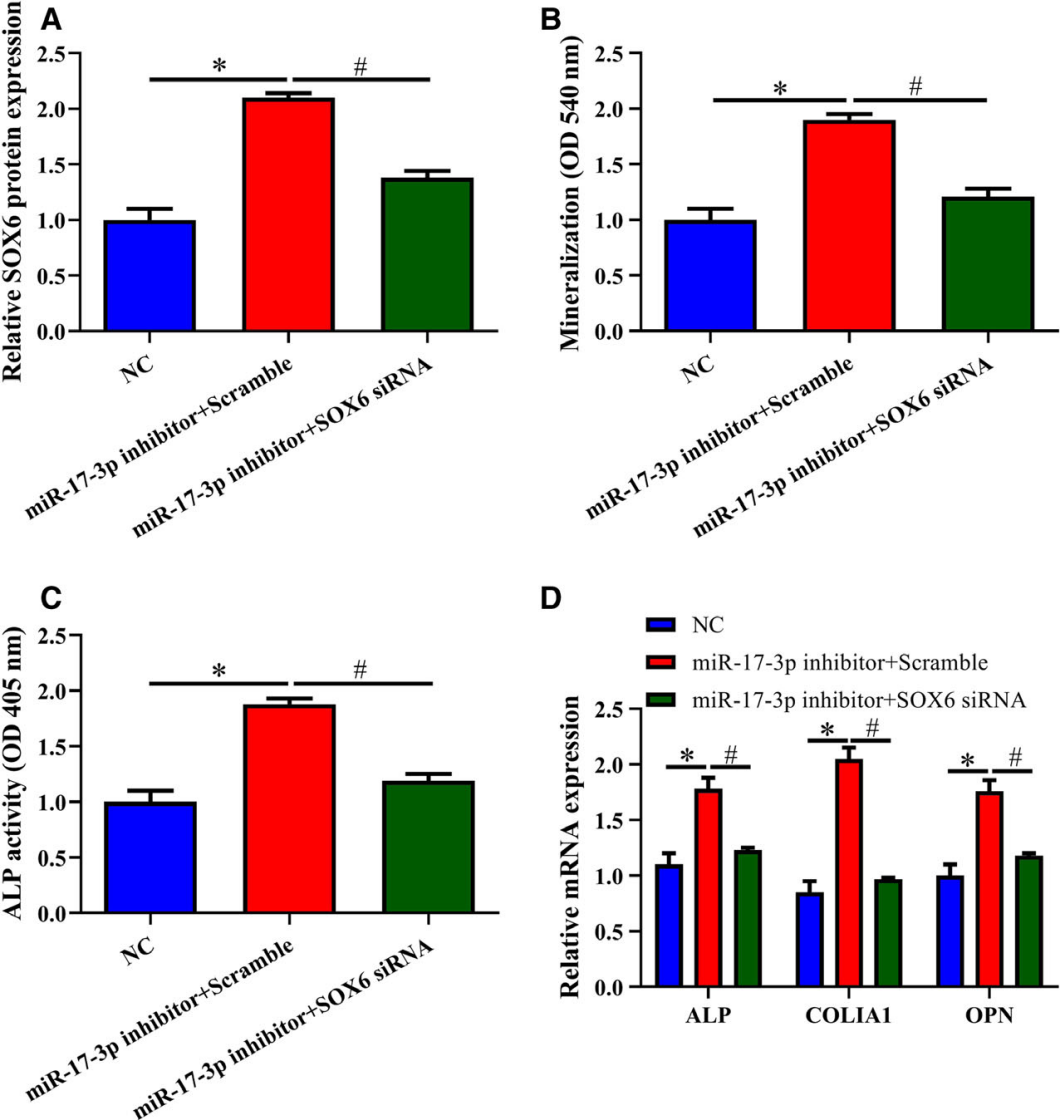
*Sox6* knockdown reverses the effects of miR‐17‐3p inhibition on osteoblast differentiation. MC3T3‐E1 cells were cotransfected with miR‐17‐3p inhibitor and *Sox6* siRNA for 24 h, followed by induction of osteoblast differentiation by BMP2 treatment for 14 days. (A) Relative expression level of Sox6 protein. (B) Matrix mineralization evaluated by Alizarin Red staining. (C) ALP activity detected with a commercial assay kit. (D) mRNA levels of osteoblast marker genes (*ALP*, *COLIA1*, and *OPN*) determined by RT‐qPCR. The error bars indicate SD. Wilcoxon test was used to evaluate the differences between groups. *n* = 3, *P < 0.05, **P < 0.01; ^#^ *P* < 0.05, ^##^ *P* < 0.01.

To examine the relationship between miR‐17‐3p and *Sox6* in greater detail, we cotransfected miR‐17‐3p inhibitor and *Sox6* siRNA into MC3T3‐E1 cells for 24 h, followed by BMP2 treatment for 14 days to induce osteoblast differentiation. *Sox6* knockdown decreased the level of *Sox6* in cells transfected with miR‐17‐3p inhibitor compared to those transfected with NC or miR‐17‐3p inhibitor + scrambled siRNA (Fig. 4A). Additionally, *Sox6* depletion reduced mineralization in the miR‐17‐3p inhibitor group compared with the miR‐17‐3p inhibitor + scrambled siRNA group (Fig. 4B). Similar trends were observed for ALP activity and *ALP*, *COLIA1*, and *OPN* mRNA levels (Fig. 4C,D). Thus, miR‐17‐3p inhibits osteoblast differentiation via negative regulation of *Sox6* expression.

## Discussion

In the present study, we observed that miR‐17‐3p expression decreased during BMP2‐induced osteoblast differentiation in MC3T3‐E1 cells. Gain‐ and loss‐of‐function experiments using miR‐17‐3p mimic or inhibitor, respectively, showed that miR‐17‐3p suppressed mineralization, ALP activity, and the expression of osteogenesis‐associated genes (*ALP*, *Col1A1*, and *OPN*). We also confirmed that miR‐17‐3p directly regulates *Sox6* expression in MC3T3‐E1 cells during osteoblast differentiation. These data provide evidence for miR‐17‐3p as a negative regulator of osteogenesis that acts by inhibiting *Sox6* and its downstream targets.

Osteoblasts are essential for maintaining the stability of the intraosseous environment. Multiple miRNAs have been identified that participate in osteoblast differentiation. For example, miR‐29b, miR‐210, miR‐335‐5p, and miR‐2861 were shown to enhance this process [15, 16, 17, 18], whereas miR‐125a‐3p, miR‐145‐5p, miR‐106b‐5p, and miR‐17‐5p exert suppressive effects [19, 20, 21]. MiR‐17‐3p is variably expressed in different cancer types [22, 23, 24] but its role in osteogenesis has not been previously reported. The MC3T3‐E1 preosteoblast cell line has been widely used for *in vitro* studies of osteogenesis. We used these cells in the present study to investigate the mechanism by which miR‐17‐3p regulates osteoblast differentiation. Interestingly, the miR‐17‐92 cluster has been shown to regulate bone growth and development; as a mature miRNA within this cluster, miR‐17‐3p has been suggested to play an essential role in bone formation [25], which is supported by the current findings.

MiRNAs mainly act by modulating the expression of target transcripts. Numerous miRNAs are known to participate in osteogenesis either as positive or negative regulators [26]. *Sox6* encodes a transcription factor that is involved in the differentiation of various cell types including mesenchymal stem cells and neurons [14, 27, 28]. OS, which is common in children and adolescents, is characterized by impaired bone formation resulting from abnormal osteogenesis [29]. Sox6 was shown to suppress proliferation, invasion, and epithelial‐to‐mesenchymal transition in OS cells by targeting TWIST1 [14]. Reduced differentiation and loss of function of osteoblasts are key features of osteoporosis and osteoarthritis. A large‐scale meta‐analysis identified Sox6 as a candidate gene that increased bone mineral density and thereby improved osteoporosis in women [30]. Sox6 and Sox9 are also important factors in cartilage homeostasis that stimulate cartilage formation, which may promote bone growth and prevent osteoarthritis [31]. In our study, we found that miR‐17‐3p suppressed osteoblast differentiation by downregulating the expression of Sox6, although the clinical significance of this observation in the context of OS or osteoporosis remains to be determined. Additionally, the detailed mechanism of *Sox6* regulation by miR‐17‐3p in osteogenesis warrants further study, although there is evidence suggesting that Sox6 specifically activates enhancers of target genes in primary osteoblasts [32].

In conclusion, the results of our study demonstrate for the first time that miR‐17‐3p negatively regulates osteoblast differentiation by suppressing *Sox6* expression. Thus, therapeutic strategies that inhibit miR‐17‐3p could potentially stimulate bone formation and may be an effective treatment for OS and orthopedic disorders such as osteoporosis and osteoarthritis.

## Conflict of interest

The authors confirm that they have no financial or non‐financial conflicts of interest.

## Author contribution

HY conceived and designed the entire study; NC and DW analyzed the data, performed literature research, and drafted the paper. HL and YL were responsible for data analysis and visualization. HY guided the entire study and revised it critically for important intellectual content. All authors have read and agreed with the final version of this manuscript.

## Data Availability

Data will be available from the corresponding author upon reasonable request.
